# Class C G protein-coupled receptors: reviving old couples with new partners

**DOI:** 10.1007/s41048-017-0036-9

**Published:** 2017-02-28

**Authors:** Thor C. Møller, David Moreno-Delgado, Jean-Philippe Pin, Julie Kniazeff

**Affiliations:** Institut de Génomique Fonctionnelle (IGF), CNRS, INSERM, Univ. Montpellier, 34094 Montpellier, France

**Keywords:** G protein-coupled receptors, Dimerization, Activation mechanism, Glutamate, GABA

## Abstract

G protein-coupled receptors (GPCRs) are key players in cell communication and are encoded by the largest family in our genome. As such, GPCRs represent the main targets in drug development programs. Sequence analysis revealed several classes of GPCRs: the class A rhodopsin-like receptors represent the majority, the class B includes the secretin-like and adhesion GPCRs, the class F includes the frizzled receptors, and the class C includes receptors for the main neurotransmitters, glutamate and GABA, and those for sweet and umami taste and calcium receptors. Class C receptors are far more complex than other GPCRs, being mandatory dimers, with each subunit being composed of several domains. In this review, we summarize our actual knowledge regarding the activation mechanism and subunit organization of class C GPCRs, and how this brings information for many other GPCRs.

## Introduction: class C GPCRs and topology

The class C of G protein-coupled receptors (GPCRs) contains 22 members, including the eight metabotropic glutamate receptors (mGlu_1–8_), the GABA_B_ receptors (GABA_B1_ and GABA_B2_), the calcium-sensing receptor (CaS) and the taste receptors (T1R1–T1R3) (Fredriksson* et al*. 2003). The mGlu receptors have been further classified into three different groups depending on their similarities in sequence, pharmacology, signalling and localization: Group I includes mGlu_1_ and mGlu_5_, Group II mGlu_2_ and mGlu_3_ and Group III mGlu_4_, mGlu_6_, mGlu_7_ and mGlu_8_.

**Fig. 1:**
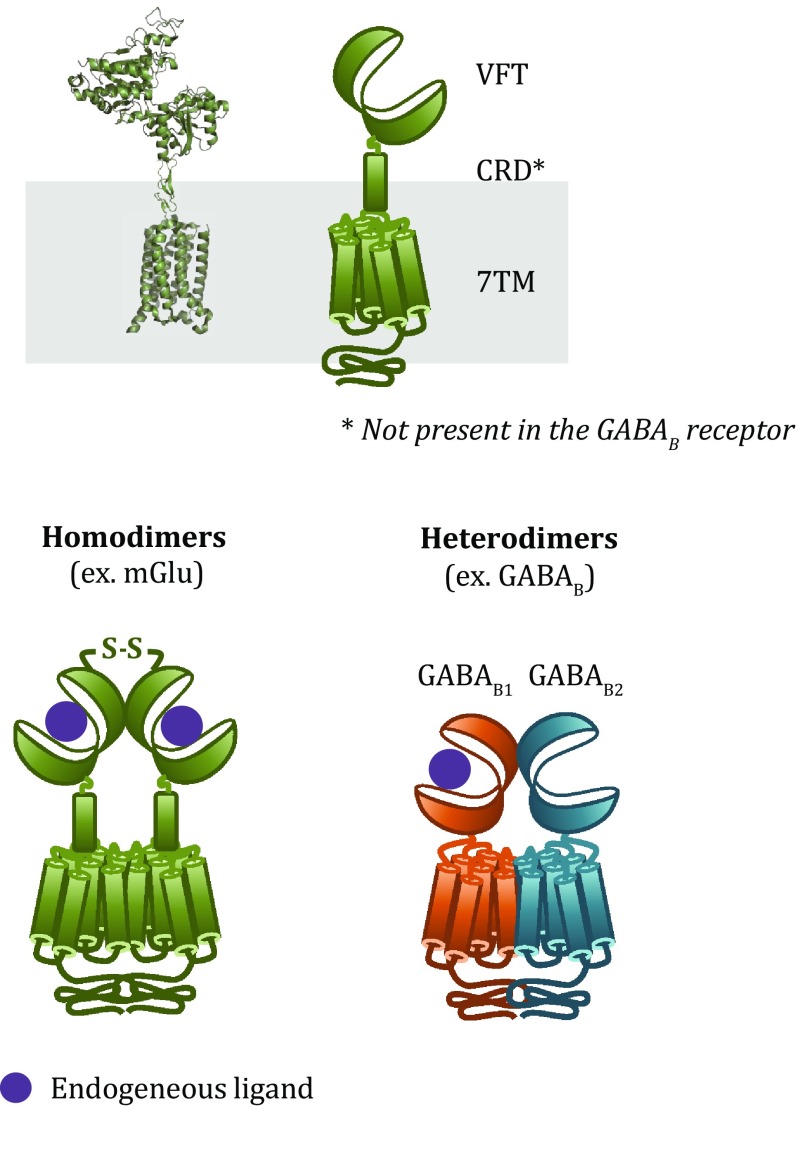
Structural model and schematic representation of class C GPCRs. Class C GPCRs are composed of a Venus flytrap (VFT) domain, a cysteine-rich domain (CRD) and a transmembrane (7TM) domain. This class of receptors forms obligatory dimers, either homodimers (*e.g*. mGlu) or heterodimers (*e.g*. GABA_B_)

Structurally, most class C GPCRs contain an extracellular so-called Venus flytrap (VFT) domain, a bilobed structure with a crevice between the two lobes that encloses the orthosteric binding site (Fig. 1). Agonist binding stabilizes a conformation with a shorter distance between the two lobes termed the closed conformation (Kunishima* et al*. 2000). The VFT domain is connected to the seven-transmembrane (7TM) domain through a cysteine-rich domain (CRD), which is notably absent in the GABA_B_ receptor (Kunishima* et al*. 2000; Muto* et al*. 2007). The 7TM domain shares similarities with other class C GPCRs both in topology and in activating similar G proteins. In addition, class C GPCRs are either homodimers (*e.g*. mGlu receptors) or heterodimers (*e.g*. GABA_B_) (Fig. 1). Here, we summarize the insights into the activation mechanism of this class of dimeric receptors gained in particular from structural and mutagenesis studies, and then we review the emerging evidence for new types of class C GPCR heterodimers or higher order oligomers.

## Dimerization of class C GPCRs: a necessity for signal transduction

### Insights from homodimers: mGlu and CaS receptors

mGlu and CaS receptors are prototypical homodimers that are stabilized by an inter-protomer disulphide bond, polar contacts between VFT domains and interactions between 7TM domains. The dimerization of these receptors is critical for promoting the activation mechanism, leading from agonist binding to G protein activation. Indeed, different studies indicate that the conformation of one protomer is relative to the other changes upon activation, and this has been observed in all the structural domains found in class C GPCRs (Pin and Bettler 2016).

**Fig. 2:**
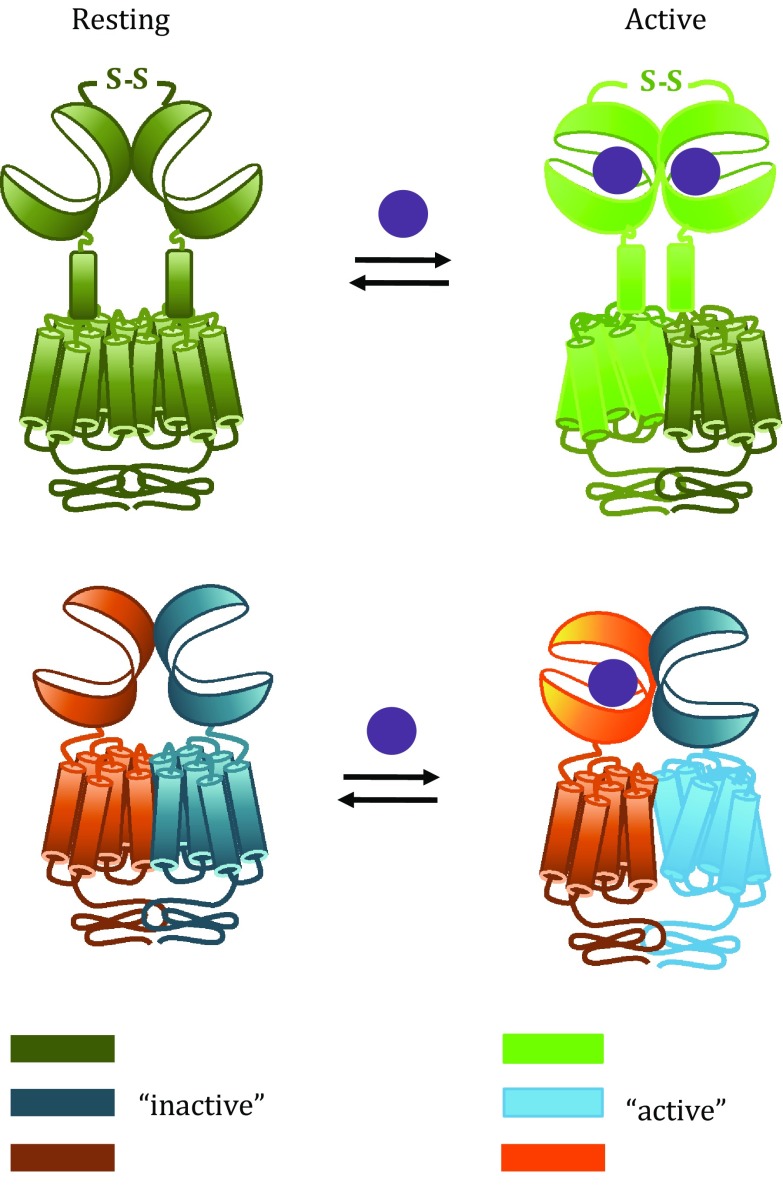
Mechanism of activation of homodimers and heterodimers. Both homodimers and heterodimers undergo conformational changes upon activation. The relative orientation of the VFT dimer is changed upon agonist binding; the CRDs (not in GABA_B_) are getting closer and the 7TM dimer changes conformation such that a single 7TM is in the active state

The dimer of VFT domains is in equilibrium between a resting and an active orientation, and agonist binding displaces the equilibrium towards the active state (Fig. 2). This reorientation is directly linked to G protein activation and, as a consequence, has been used to design a FRET-based sensor to monitor the receptor activation (Doumazane* et al*. 2013). Recently, single-molecule analyses using either the isolated dimer of VFT domains or the full-length mGlu_2_ receptor dimer have confirmed that the VFT domains oscillate rapidly between the resting and active orientations (Olofsson* et al*. 2014; Vafabakhsh* et al*. 2015). In addition, the studies revealed that agonists with different efficacies diverge in their ability to shift the conformational equilibrium towards the fully active state, rather than stabilizing intermediate conformations.

Because reorientation of the VFT domain dimer is tightly linked to G protein activation, it is implied that this conformational change is somehow transmitted to the 7TM domains. The CRD has been shown to play a critical role in this process for mGlu receptors. This domain is highly rigid due to four intramolecular disulphide bonds (Muto* et al*. 2007), and disrupting them by mutagenesis impairs the capacity of orthosteric agonists to activate G proteins (Huang* et al*. 2011). In addition, crosslinking the two CRDs in a dimer results in a constitutively active receptor (Huang* et al*. 2011). Altogether, this indicates that the transmission from VFT to 7TM domain is mediated by the rigid CRDs coming into close proximity (Fig. 2).

At the 7TM domain level, activation of the receptor requires a rearrangement of the interface between the 7TM domains in the dimer. Actually, it has been shown for the mGlu_2_ receptor that this interface in the inactive state is formed by transmembrane helix 4 (TM4) and TM5 in each protomer, while the two TM6 s are facing each other in the active state (Xue* et al*. 2015). This major change in the dimer interface is required for receptor activity, demonstrated by locking the TM4–TM5 interface, which prevents activation by agonist, and locking the TM6 interface, which leads to a constitutively active receptor (Xue* et al*. 2015). However, the crystal structure of the mGlu_1_ receptor 7TM domain in the presence of a negative allosteric modulator (NAM) suggested an alternative dimerization interface involving TM1 (Wu* et al*. 2014). While this difference might be attributed to crystal packing or lack of the VFT domain, further studies are required to determine whether a common mechanism describing the movement of the 7TM domain dimer can be defined for all class C GPCRs.

The data mentioned above suggest that the activation of mGlu receptors relies on the conformational changes of one protomer relative to the other one, which underlies the strict requirement of the mGlu dimerization for glutamate to activate G proteins (Fig. 2). This is further confirmed by experiments showing that glutamate fails to activate an isolated mGlu monomer reconstituted in nanodiscs whereas it activates an mGlu dimer (El Moustaine* et al*. 2012).

Several studies indicate that a single 7TM domain reaches the active conformation in an mGlu homodimer (Goudet* et al*. 2005; Hlavackova* et al*. 2005). In addition, an isolated mGlu monomer purified and reconstituted in nanodiscs activates G proteins when stimulated by a positive allosteric modulator (PAM) (El Moustaine* et al*. 2012). Hence, in the context of a class C GPCR homodimer, G proteins might be activated through the ligand-bound subunit (*cis*-activation) and/or through the other subunit (*trans*-activation). It has been shown for the mGlu_5_ receptor that Gq protein can be activated either by *cis*- or *trans*-activation (Brock* et al*. 2007). However, it is also possible that in some cases, depending on whether the receptor is *cis*- or *trans*-activated, the pathways activated may differ. For instance, it has been observed using a combination of glutamate binding-deficient and G protein coupling-deficient receptors that the mGlu_1_ receptor triggers Gq-coupled signalling through *cis*- and *trans*-activation, while Gi/o and Gs are exclusively activated through *cis*-activation (Tateyama and Kubo 2011).

Another point to acknowledge when considering class C GPCR homodimers is cooperativity. It has been observed that although glutamate binding to one protomer could induce receptor activation, binding to both protomers was required for full activity (Kniazeff* et al*. 2004). In addition, binding to one protomer can induce negative cooperativity to the second protomer (Suzuki* et al*. 2004), which suggests additional complexity in mGlu receptor pharmacology.

Altogether, in class C GPCR homodimers, G protein activation can be achieved upon binding of a single agonist and by a single protomer with an active 7TM domain. However, the dimeric structure is an absolute prerequisite for the conformational transitions from endogenous agonist binding to G protein activation and thus for physiological receptor function.

### Insights from heterodimers: GABA_B_ and T1Rs

The GABA_B_, sweet taste and umami taste receptors are prototypical class C heterodimers: two different subunits are required to activate G proteins upon agonist binding, confirmed *in vivo* for the GABA_B_ receptor by the disappearance of all physiological responses attributed to the heterodimer when either of the two subunits is knocked out (Prosser* et al*. 2001; Schuler* et al*. 2001; Queva* et al*. 2003; Zhao* et al*. 2003; Gassmann* et al*. 2004). The GABA_B_ receptor is a non-covalently linked obligatory heterodimer composed of the subunits GABA_B1_ and GABA_B2_ (Jones* et al*. 1998; Kaupmann* et al*. 1998; White* et al*. 1998), while the taste receptors are composed of T1R3 and either T1R1 or T1R2 resulting in umami or sweet taste receptors, respectively (Nelson* et al*. 2001, 2002) (Fig. 1). For the GABA_B_ receptor, the GABA_B1_ subunit contains the binding site for orthosteric ligands (Galvez* et al*. 1999, 2000), while the GABA_B2_ subunit is necessary for G protein activation (Margeta-Mitrovic* et al*. 2001a; Robbins* et al*. 2001; Duthey* et al*. 2002), confirming the absolute necessity of heterodimer formation. In contrast, the T1R2 subunit seems to be responsible for binding of most ligands and for G protein activation in the sweet taste receptor (Xu* et al*. 2004). Interestingly, the attempt to create a homomeric receptor by fusing the ligand binding and the G protein coupling domains from GABA_B_ resulted in a non-functional receptor (Galvez* et al*. 2001), indicating a unique activation mechanism for these heterodimeric receptors.

In the GABA_B_ receptor, the correct assembly of the heterodimer is ensured by the C-terminal tail: when expressed alone, GABA_B1_ is retained in the ER due to a RSRR retention motif located in its C-terminal tail (Couve* et al*. 1998; Margeta-Mitrovic* et al*. 2000; Pagano* et al*. 2001). In the presence of GABA_B2,_ the retention motif is masked by a coiled-coil interaction between the subunits, thus ensuring that only correctly assembled heterodimers are trafficked to the cell surface (Margeta-Mitrovic* et al*. 2000; Pagano* et al*. 2001). However, functional GABA_B_ receptors can assemble on the cell surface independently of the coiled-coil domains (Pagano* et al*. 2001). In addition to the coiled-coil domain interaction, the GABA_B_ subunits form interactions between the VFT domains (Geng* et al*. 2013) and most likely also the 7TM domains. Crystal structures of the GABA_B_ VFT dimer show that in the resting state, they interact exclusively *via* a tight interface involving only one lobe, whereas in the active state a reorientation of the GABA_B1_ VFT domain facilitates an additional, looser interaction between the second lobes (Geng* et al*. 2013).

During activation, agonists bind to the VFT domain of one protomer and promote active conformation of the 7TM domain of the other protomer (Galvez* et al*. 2001; Margeta-Mitrovic* et al*. 2001b), implying that *trans*-activation is the main process for G protein activation in these heterodimers (Fig. 2). Compared to homodimers, this results from a slightly different mechanism. For example, in the GABA_B_ receptor, the GABA_B2_ VFT domain is unable to bind ligands (Kniazeff* et al*. 2002) and its closed conformation is not necessary for full activation (Geng* et al*. 2012). Total deletion of the GABA_B2_ VFT domain results in a functional receptor suggesting that the signal can proceed from the GABA_B1_ VFT domain to the GABA_B2_ 7TM domain through the GABA_B1_ 7TM domain (Monnier* et al*. 2011). On the other hand, replacing the GABA_B1_ 7TM domain by a single transmembrane helix also produced a functional receptor, suggesting that the signal may also be transmitted through the GABA_B2_ VFT domain (Monnier* et al*. 2011). Altogether, these data propose that two ways of activation exist in the GABA_B_ receptor.

Cooperativity between protomers exists also in class C GPCR heterodimers. It has been shown in the GABA_B_ receptor that although the GABA_B2_ VFT and the GABA_B1_ 7TM domains are not directly involved in ligand binding and G protein activation, they play a key role in defining the activation potency. Indeed, when expressed alone, GABA_B1_ exhibits low-affinity agonists binding; however, when co-expressed with GABA_B2,_ the interaction with the GABA_B2_ VFT domain increases agonist affinity tenfold (Kaupmann* et al*. 1998). Along the same lines, the GABA_B1_ 7TM domain improves the G protein coupling efficiency of GABA_B2_ (Galvez* et al*. 2001).

Altogether, in class C GPCR heterodimers, such as the GABA_B_ receptor, one subunit contains the ligand binding domain, but the other subunit is critical for high-affinity agonist binding and functional responses. For the GABA_B_ receptor, the necessity of dimerization and allosteric transition is ensured by specific targeting of the heterodimer to the cell surface.

## New folks in class C GPCR oligomers

### Heterodimers of mGlu receptors

In addition to homodimers, mGlu receptors have recently been reported to possibly form eleven different heterodimers in heterologous systems: the mGlu_1_ and mGlu_5_ receptors can only heteromerize between them, whereas all combinations are possible among five other mGlu receptors (mGlu_2_, mGlu_3_, mGlu_4_, mGlu_7_ and mGlu_8_) (Doumazane* et al*. 2011). These various combinations are likely strict heterodimers and not the result of association of two homodimers as well demonstrated for the mGlu_2_–mGlu_4_ combination (Yin* et al*. 2014; Niswender* et al*. 2016). Interestingly, all possible combinations were found between receptors that share neuronal localization and G protein coupling, which suggests that heterodimer formation is not an artefact of receptor co-expression, but a specific process controlled by structural and functional properties of the receptors.

The study of mGlu heterodimers is a difficult issue to address due to two main points: the lack of specific ligands (especially for receptors from the same group) and the presence of both homodimers and heterodimers in cells co-expressing two mGlu receptors (Doumazane* et al*. 2011). However, some studies have tried to address the topic focusing on heterodimers between mGlu_2_ and mGlu_4_ receptors. Although the precise function and pharmacological properties of these heterodimers in native tissues remain open questions, mGlu_2_ and mGlu_4_ receptors were found to co-immunoprecipitate in rat dorsal striatum and medial prefrontal cortex (Yin* et al*. 2014). Regarding orthosteric agonist activation, partial agonists such as DCG-IV seem to have a reduced effect in heterodimer activation in comparison with mGlu_2_ homodimers (Kammermeier 2012), and full agonists such as LY379268 seem to be less potent in activating mGlu_2_–mGlu_4_ heterodimers and exhibit dose–response curves with reduced slope (Yin* et al*. 2014). Regarding heterodimer activation by PAMs, mGlu_2_ PAMs do not activate the heterodimer (Kammermeier 2012), whereas the effect of mGlu_4_ PAMs depends strongly on their scaffold: VU0155041 seems to activate the heterodimer, but not PHCCC (Yin* et al*. 2014) or VU0418506 (Niswender* et al*. 2016). Further research in the binding site of these PAMs could lead to compounds activating only mGlu_2/4_ heterodimers, which could help to understand their physiological role.

### Higher order GABA_B_ oligomers

The existence of higher order oligomers of GPCRs is still a topic open for discussion, especially because most of the observations have been done in heterologous cells and not validated in native tissues (Vischer* et al*. 2015). However, increasing experimental evidence suggests that the GABA_B_ receptor forms oligomers larger than heterodimers. First, in time-resolved FRET experiments, a strong FRET signal was measured between GABA_B1_ subunits, whereas the signal between GABA_B2_ subunits was weak. This led to the proposal that the GABA_B_ receptor forms at least dimers of heterodimers associated through the GABA_B1_ subunits (Maurel* et al*. 2008; Comps-Agrar* et al*. 2011, 2012). Notably, the FRET/receptor ratio was constant over a wide range of receptor densities, including expression levels similar to the endogenous levels in the brain (Maurel* et al*. 2008). In addition, the existence of oligomers larger than tetramers was suggested by single-molecule microscopy experiments in CHO cells, which showed that at low densities, the majority of GABA_B_ receptors were dimers with a smaller population (~30%) of tetramers, whereas at higher densities the dimer population disappeared and complexes larger than tetramers appeared, representing ~60% at the highest density (Calebiro* et al*. 2013).

In native tissues, the existence of GABA_B_ oligomers is more complex to prove, but some evidence supports the proposal. Indeed, a FRET signal between anti-GABA_B1a_ antibodies was detected in brain membrane from wild-type animals, but not from GABA_B1a_ knockout animals (Comps-Agrar* et al*. 2011), and the migration of GABA_B_ receptors from brain membranes on native gels is consistent with complexes larger than dimers (Schwenk* et al*. 2010).

A possible function of the oligomerization of the GABA_B_ heterodimer is modulation of receptor signalling. Indeed, it was found that inhibiting GABA_B1_–GABA_B1_ interactions using either a non-functional GABA_B1_ subunit as competitor or introducing a mutation in the GABA_B1_ VFT domain increased signalling efficacy by approximately 50% (Maurel* et al*. 2008; Comps-Agrar* et al*. 2011). It was further shown that one ligand or one G protein per oligomer was sufficient to achieve full activation, suggesting negative cooperativity between heterodimers (Comps-Agrar* et al*. 2011).

## Conclusions

Class C GPCRs are acknowledged to be dimeric. Over the last two decades, an increasing number of studies have shed light on the necessity of this dimerization for their mechanism of activation. These studies also proposed general concepts for the activation of GPCR dimers. In recent years, new combinations of class C GPCRs with specific pharmacological properties have been reported in heterologous systems and may reveal an even higher complexity of the glutamatergic and GABAergic modulation of the synaptic activity.

## Abbreviations

7TM domain: Seven-transmembrane domain
CaS receptor: Calcium-sensing receptor
CRD: Cysteine-rich domain
GABA: γ-Aminobutyric acid
GPCR: G protein-coupled receptor
mGlu receptor: Metabotropic glutamate receptor
NAM: Negative allosteric modulator
PAM: Positive allosteric modulator
TM: Transmembrane
VFT: Venus flytrap
